# Mineralization and nutrient release pattern of vermicast-sawdust mixed media with or without addition of *Trichoderma viride*

**DOI:** 10.1371/journal.pone.0254188

**Published:** 2021-07-08

**Authors:** Suwen Lin, Lokanadha Rao Gunupuru, Raphael Ofoe, Roksana Saleh, Samuel Kwaku Asiedu, Raymond H. Thomas, Lord Abbey

**Affiliations:** 1 Department of Plant, Food, and Environmental Sciences, Faculty of Agriculture, Dalhousie University, Truro, Nova Scotia, Canada; 2 Department of Environmental Science and Boreal Ecosystem Research Initiative, Memorial University of Newfoundland, Corner Brook, Newfoundland and Labrador, Canada; Banaras Hindu University, INDIA

## Abstract

A combination of vermicast and sawdust mixed medium is commonly used in horticulture, but the added benefit of microbial inoculation and mechanism of nutrient availability are unknown. This study was done to determine nutrient mineralization and nutrient release patterns of different combinations or a mix of vermicast-sawdust growing media amended with or without *Trichoderma viride* (10^5^ spores/g). The mixed-media treatments were (1) 80% vermicast+20% sawdust; (2) 60% vermicast+40% sawdust; (3) 40% vermicast+60% sawdust; (4) 20% vermicast+80% sawdust; and (5) sawdust alone (control). Total dissolved solids, electric conductivity and salinity increased with each sampling time following submergence in deionized. Nutrients released from media without *T*. *viride* were significantly higher than the corresponding media with added *T*. *viride*. Overall, the starting total nitrogen of the different media did not change during the incubation period, but nitrate-nitrogen was reduced to a negligible amount by the end of day 30 of incubation. A repeated measures analysis showed a significant effect of Time*T. viride*Treatment on total dissolved solids. Redundancy analysis demonstrated a positive and strong association between media composed of ≥40% vermicast and ≤60% sawdust with or without *T*. *viride* and mineral nutrients released, electrical conductivity, total dissolved solids and salinity. These findings suggest that fast-growing plants may benefit from 40% to 60% vermicast added to 40% to 60% sawdust without *T*. *viride* while slow-growing plants can benefit from the same mixed medium combined with the addition of *T*. *viride*. Further investigation is underway to assess microbial dynamics in the mixed media and their influence on plant growth.

## Introduction

Plant growth and development are largely dependent on available nutrients, temperature, heat, water, pests and diseases incidences, which may directly or indirectly influence plant uptake and utilization of nutrients. Plants obtain essential mineral elements for their growth from the soil, organic amendments or synthetic chemical fertilizers [1, 2]. The availability of these mineral elements and their uptake efficiency by plants is influenced by several biological and biochemical processes including biotransformation through mineralization or immobilization and fixation [3]. Nutrients can also be immobilized or rendered unavailable due to microbial use, chelation and interference with other competing nutrients through cation or anionic exchange processes [3, 4]. For instance, a C/N ratio ≥ 30:1 suggests immobilization of mineral N by decomposing microbes that absorb mineral N in the form of ammonium or nitrates leading to reductions in available N for plant uptake [3, 5]. Moreover, nutrient availability and mineralization efficiency are increased when its concentration exceeds the needs of microbial decomposers for biosynthesis or storage [6].

In sustainable agricultural systems, natural amendments such as vermicast, a pure worm excreta, is widely used to improve the quality and health of growing medium and to meet the nutritional needs of plants [7]. A greenhouse pot experiment revealed that application of vermicast alone (100%) or 75% vermicast with added *Mycorrhizal* fungi were toxic to plants due to high chemical nutrients concentration compared to the addition of 25% or 50% vermicast [8]. Vermicast is rich in beneficial microorganisms, essential nutrients, humic and non-humic substances and growth-promoting hormones with desirable physical properties [9–11]. Additionally, the large surface area of vermicast granules provides more microsites for microbial activity and nutrients retention [12, 13], which could lead to slow nutrient release for an extensive period. However, literature information on vermicast nutrient mineralization and pattern of release over a pre-determined period is limited. The understanding of these patterns is important if vermicast is to be used extensively in sustainable agricultural systems as a nutrient supplement for plants.

The use of sawdust as a growing medium substrate has attracted the interest of many researchers and the greenhouse industry due to its ability to improve growing medium properties such as enhanced porosity and water retention of the growing medium [14]. However, the high C/N ratio and potential toxic compounds could adversely affect microbial community activities, nutrients availability and plant growth. These effects can be reduced by thermal treatment of sawdust which involves heating wood to high temperatures between 160°-250°C. For instance, the content of potentially phytotoxic hemicellulosic sugar i.e., arabinose, xylose, mannose and galactose in oak (*Quercus* spp.) sawdust was reduced by thermal treatment compared with non-thermally treated sawdust, which has higher glucose, P, K, Ca and Mg contents [15]. Additionally, thermally treated oak sawdust had higher porosity and water-holding capacity than non-thermally treated samples. Thermal treatment of sawdust also reduced lignin, hemicellulose and cellulose crystallinity. Earlier studies revealed that foliar application of untreated sawdust mixed with vermicast extract (1:10 v/v; 1000 mg/l) increased N uptake in *Syngonium* spp. [16]. These findings suggested that an appropriate proportion of vermicast-sawdust mixed medium can significantly improve growing medium biological, physical and chemical properties.

*Trichoderma viride* is a free-living fungus that has beneficial association with organic and inorganic compounds in the media including humic substances found in vermicast [17]. *T*. *viride* is ubiquitous in different soils and root ecosystems and has the potential to improve growing medium nutrients availability by mineralization of carbon and nitrogen during composting and, to protect plants from pathogens by activating immunity-mediated genes through the canonical defence signals i.e., jasmonic acid and salicylic acid pathways [18]. However, the significance of *T*. *viride* abundance in a sawdust based growing medium has not been reported. Therefore, we postulate that vermicast-*Trichoderma*-sawdust mixed medium can be a holistic approach to provide essential plant nutrients and increase plant resilience to pathogens. Abbey et al. [19] determined nutrient-release from dehydrated vermicast using water quality indices i.e., electrical conductivity (EC), total dissolved solids (TDS) and salinity. Based on our preliminary study, we hypothesized that vermicast-sawdust mix media with the addition of *T*. *viride* will enhance mineralization and nutrient availability. Therefore, the objective of this study was to determine mineralization and nutrient release patterns in different proportions of vermicast-sawdust media with or without the addition of *T*. *viride*.

## Materials and methods

### Experimental location and materials

Mineralization and nutrient release studies were performed in the Compost and Biostimulant Laboratory in the Department of Plant, Food, and Environmental Sciences, Dalhousie University, Faculty of Agriculture, Truro, Canada from March 2018 to September 2019. Vermicast is the waste product produced by red wiggler worms (*Eisenia fetida*) was obtained from Pagonis Live Bait, ON, Canada; and thermally treated (>120°C and 100% relative humidity) oak (*Quercus* spp.), maple (*Acer* spp.) and aspen (*Populus tremuloides*) tree sawdust were supplied by Thermal Wood Canada, NB, Canada. Promix-BX (Premier Horticulture Inc., Quakertown, USA), a general-purpose peat-based substrate consisting of 75–85% sphagnum peat moss, horticultural-grade perlite and vermiculite, dolomitic and calcitic limestone, a wetting agent was purchased from Halifax Seed Inc., Halifax, NS, Canada. *T*. *viride* was isolated from municipal solid waste obtained from Fundy compost, Nova Scotia, Canada and characterized by ITS region gene sequencing.

### Experimental design and media preparation

The experiment was arranged in a 5×2 factorial completely randomized design with three replications. The two factors consisted of: (1) five levels of vermicast-sawdust mixed media (weight/weight): (i) 80% vermicast + 20% sawdust, (ii) 60% vermicast + 40% sawdust, (iii) 40% vermicast + 60% sawdust, (iv) 20% vermicast + 80% sawdust, and (v) sawdust alone (control); and (2) two levels of *T*. viride: (i) without (A) and (ii) with 10^5^ spores/g of *T*. *viride* (B). The fungal inoculum was prepared by adding 5 ml of pure cultured *T*. *viride* to 400 ml of sterilized deionized water and thoroughly mixed. The treatment combinations were coded as A1-A5 and B1-B5 as presented in Table 1.

**Table 1:** Mixed proportions of vermicast, sawdust and *Trichoderma viride* growing media.

Growing medium code	Vermicast (%)	Sawdust (%)	*T*. *viride*
**A1**^a^	80	20	Absent
**A2**	60	40	Absent
**A3**	40	60	Absent
**A4**	20	80	Absent
**A5**	0	100	Absent
**B1**^b^	80	20	Present
**B2**	60	40	Present
**B3**	40	60	Present
**B4**	20	80	Present
**B5**	0	100	Present

^a^A is Growing media with no *T*. *viride*.

^b^B is growing media with *T*. *viride*.

### Media incubation and nutrient mineralization

The individual growing media was incubated in the dark at room temperature for 90 days. Aliquots (200 g) of samples from each media was taken at the beginning of day 1 (i.e., just before incubation and denoted as time 0), this was followed by another sampling on 30, 60- and 90-days post-incubation. The samples were further coded as A1-1 to A5-4 and B1-1 to B5-4. A1-1 or B1-1 represented sample A1 or B1 taken on the first sampling point (i.e., beginning of incubation), and A5-4 or B5-4 represented A5 or B5 on the 4^th^ sampling point (i.e., 90 days after incubation). A total of 160 samples of mixed media were collected every 30 days during the incubation period and kept in a plastic bag, sealed and stored in a -20°C freezer (Whirlpool, Mississauga, ON, CA) to conduct the mineral nutrients analyses. The frozen samples were thawed at room temperature for 24 hr and treated as saturated greenhouse soil paste according to standard laboratory protocol [20] of the Nova Scotia Department of Agriculture Laboratory Services in Truro, NS. Complete micro-and macro-nutrients including total N, nitrate (NO_3_^-^), phosphorus (P), potassium (K), calcium (Ca), magnesium (Mg), boron (B), iron (Fe), manganese (Mn), copper (Cu), zinc (Zn), sodium (Na), chlorine (Cl), sulphate (SO_4_^2-^) and aluminum (Al) were analyzed using the AOAC-968.08 inductively coupled plasma mass spectrometer (ICP-MS) method [20].

### Nutrient release

The nutrient release method described by Abbey et al. [19] with some modification was used. Briefly, 20-g samples of each mixed media were placed in a rosin press nylon bag (i.e., 2.5 cm x 10.2 cm of mesh-size 160 μm) and submerged into a clean glass column (i.e., 35-cm height and 10-cm inner diameter) containing 500 ml deionized water. The bags were weighed down with washed pebbles. To determine nutrients released into the deionized water, triplicate samples of the nutrient solution was collected in a 2.5-cm depth sample cup and total dissolved solids (TDS), electric conductivity (EC), salinity and pH were measured using ExStik^®^ EC500 instrument (Extech Instruments Corporation, NH, USA). These chemical indices were recorded every 15 min up to 30 min after submergence; followed by every 30 min up to 1.5 hr; every 1 hr up to 3 hr; 3 hr; 5.5 hr; 9 hr; 12 hr; and then every 24 hr for 7 days.

### Statistical analysis

All data collected were subjected to two-way analysis of variance (ANOVA) using Minitab version 19.1 (Minitab Inc., PA, USA). Treatment means were separated and compared using Fisher’s least significant difference (LSD) method at α = 0.05. Student t-test was also used to compare media quality among the two groups i.e., media without (A) versus media with added *T*. *viride* (B). A nonlinear regression model was used to explain the pattern of nutrient release. The nonlinear regression model was:

$$y_{i}=f\left(x_{i},\theta \right)+\varepsilon_{i};$$

where θ represents a parameter vector that has more than one value (θ1, θ2, θ3,…, θ*k*); y_i_ is each response i.e., i = 1, … n; where n and x_i_ represent the two independent variables. Repeated measures analysis was also carried out to examine simple factor effects (main effects) and interaction effects, and to reduce variability among treatments. All statistical analyses were performed using Minitab and two-dimensional redundancy analysis using XLSTAT version 19.1 (Addinsoft, NY, USA).

## Results and discussion

**Fig 1:**
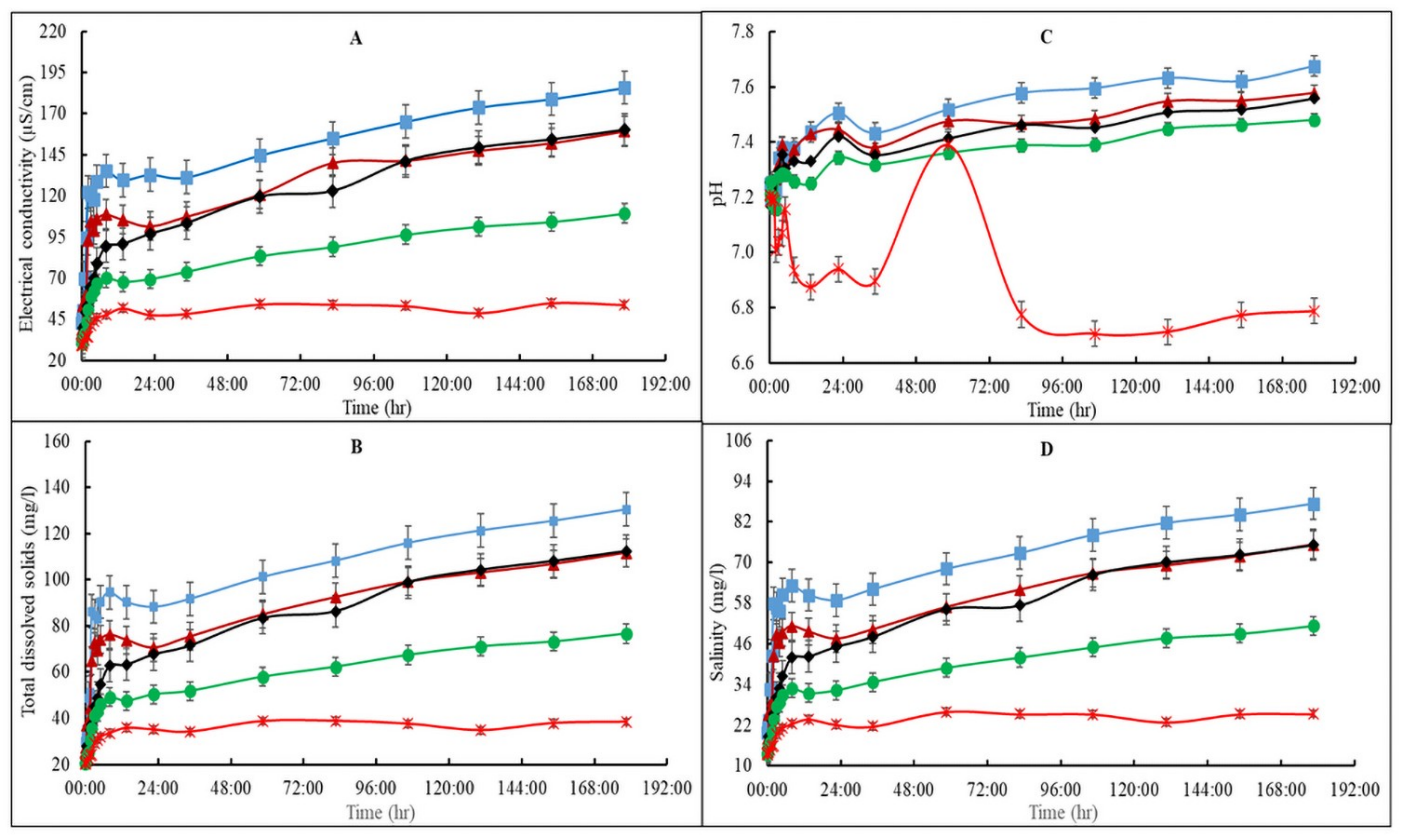
Electric conductivity (A), total dissolved solids (B), salinity (C) and pH (D) of vermicast-sawdust mixed media without addition of *Trichoderma viride* (group A). A1 (blue line) is 80%vermicast+20%sawdust; A2 (brown line) is 60%vermicast+40%sawdust; A3 (black line) is 40%vermicast+60%sawdust; A4 (green line) is 20%vermicast+ 80%sawdust; and A5 (red line) is sawdust alone with n = 3 per experimental replicate. Error bars represent standard error.

Group A media (i.e., A1—A5) with no *T*. *viride* showed rapid initial release of nutrients as determined by the EC, TDS and salinity of the deionized water (Fig 1A–1C). The EC, TDS and salinity for A1—A4 rose steadily up to the 8^th^-hr followed by a rapid decline. The water quality parameters increased sharply again and maintained a gentle rise to 178.5 hrs except for A5, which levelled off after the first 8 hrs (Fig 1A–1C). However, the EC, TDS and salinity of group A growing media followed similar patterns i.e., A1 > A2 = A3 > A4 > A5 (Fig 1A–1C).

These similar patterns in water quality properties can be attributed to the existing relationship among TDS, EC and salinity. Besides, the pH of the deionized water rose sharply from the beginning up to the 22.5^th^-hr for A1 and A2 followed by a sudden dip at the 34.5^th^- hr. For A3 and A4, the pH of the deionized water dipped at the 13.5^th^-hr before rising for 9 hr and dipping again at the 34.5^th^-hr. All the pH values for treatments A1—A4 rose gently after the 34.5^th^-hr. On the contrary, the pH of the sawdust alone treatment (A5) fluctuated for the first 85 hr with a sharp rise from a pH of 6.8 to a peak pH of 7.4 at the 58.5^th^-hr. The pH of A5 then dropped significantly by approximately 10% at the 96^th^-hr where it remained constant until the end of the experiment (Fig 1D). These variations can be ascribed to high hydrogen ion concentration in the sawdust alone, which made the solution more acidic compared to a mixture of sawdust and vermicast medium [21]. Overall, the pattern for pH was A1 > A2 > A3 = A4 >A5, which was like the patterns for the TDS, EC and salinity. This pattern can be positively associated with variations in the proportion of vermicast in the mixed media. The higher the proportion of vermicast, the higher the values of the four water quality parameters measured in the mixed media combinations.

**Fig 2:**
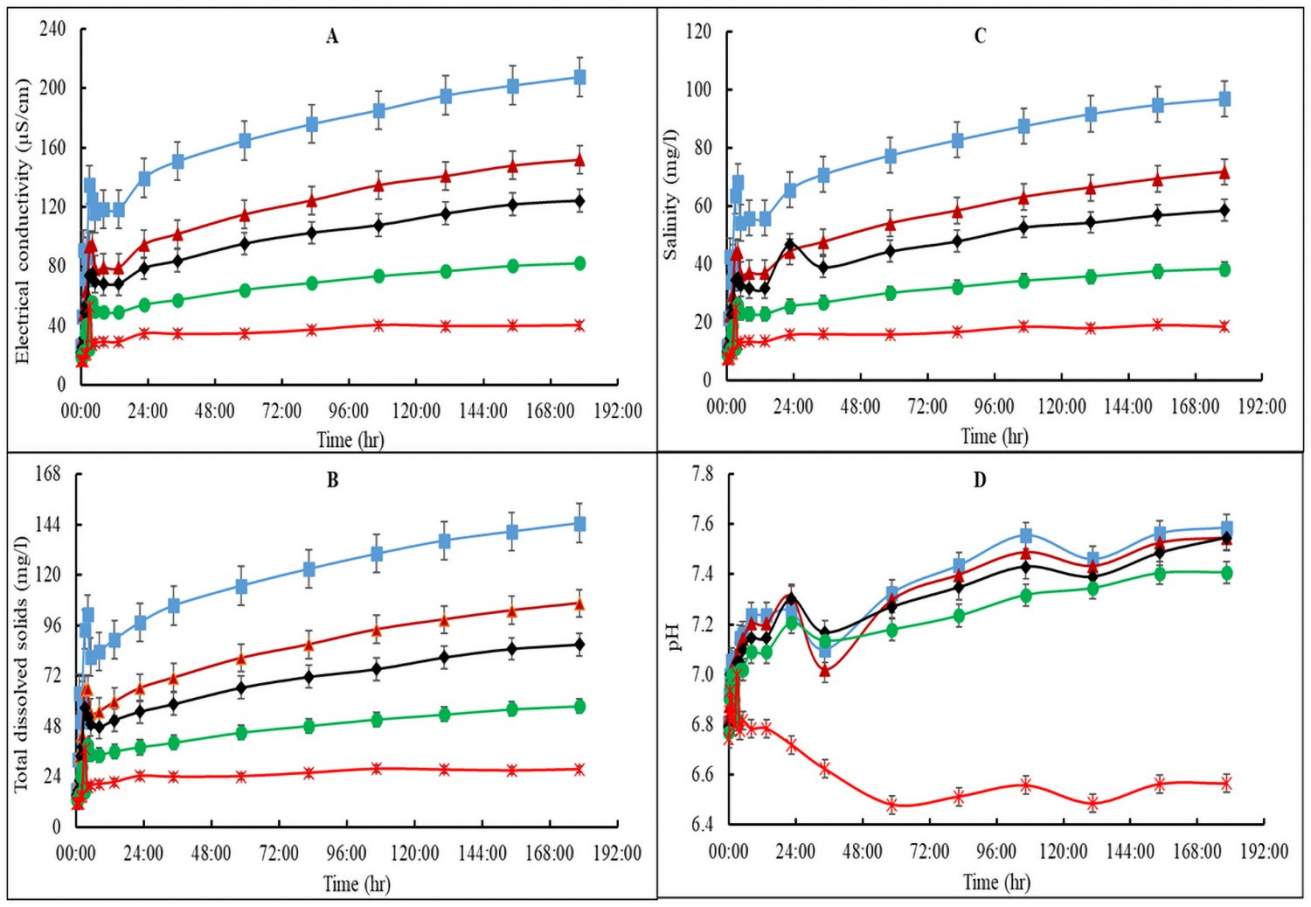
Electric conductivity (A), total dissolved solids (B), salinity (C) and pH (D) of vermicast-sawdust mixed media with addition of *Trichoderma viride* (group B). B1 (blue line) is 80%vermicast+20%sawdust; B2 (brown line) is 60%vermicast+40%sawdust; B3 (black line) is 40%vermicast+60%sawdust; B4 (green line) is 20%vermicast+ 80%sawdust; and B5 (red line) is sawdust alone with n = 3 per experimental replicate. Error bars represent standard error.

The overall pattern of the EC, TDS and salinity for treatment B was somewhat like that of treatment A except for treatment B3, which was different from treatment B4 (Fig 2A–2C) unlike A3, which was similar to A4 (Fig 1A–1C). Moreover, the initial nutrient release activities for treatment B were different from that of treatment A. Overall, EC, TDS, and salinity levels of treatment B increased sharply from the beginning up to the 3^rd^-hr before declining for the next 3 hr after which it rose again gently and remained at a constant rate from the 13.5^th^-hr until the end of the experiment. The pattern for treatment B water quality parameters was B1 > B2 > B3 > B4 > B5. Comparatively, A1 and B1 recorded the highest EC, TDS and salinity values, which also corresponded to the highest proportion of added vermicast. It was also found that nutrient release measured by the water quality parameters were higher for treatment A media than those of treatment B media (Figs 1A–1C and 2A–2C). Therefore, the EC, TDS and salinity values suggested that the addition of *T*. *viride* reduced media nutrient release. The individual media within each group also differed in their nutrient release characteristics except for A3 and A4, which were similar.

The pH for all the *T*. *viride* amended growing media of treatment B (Fig 2D) followed a similar trend and fell within the range of values for treatment A media, except for the obvious fluctuations prior to the 82.5^th^-hr (Fig 1D). Nevertheless, the pH of B5 was consistently reduced until it stabilized after 58 hrs. The pH of B1—B4 increased sharply before declining at the 22^nd^-hr but rose gently again with minimum fluctuations after 12 hr. The pattern of treatment B media pH was B1 = B2 = B3 > B4 > B5 (Fig 2D). Overall, the rate of change in water quality indices for treatment B was consistently slower compared to those of treatment A. This can be attributed to the presence of *T*. *viride* in treatment B growing media which for some reason, reduced the rate of nutrient released into solution. For most plants, the optimum pH is between 6.0 and 7.8. Usually, 65% of the applied nutrients are available for plants at growing medium pH levels below 5.9, while 35% may be available below pH of 5.5 [22]. As such, all the mixed media except treatment A5 and B5 were suitable for growing most plants. Consistently, pH was not altered by the addition of *T*. *viride* in vermicast amended media since vermicast is capable of buffering growing medium pH [23, 24]. Without vermicast, the pH of treatment A5 was found to be moderately higher than that of B5.

An independent 2-sample t-test analysis revealed no significant (P>0.05) differences amongst EC, TDS and salinity values between A1 versus B1, A2 versus B2 or A3 versus B3 (Table 2). However, there was a non-significant (P>0.05) pattern of B1 > A1, A2 > B2 and A3 > B3. On the other hand, EC, TDS and salinity for A4 and A5 were all increased significantly (P<0.05) by an average of 36% and 46.7% compared to B4 and B5, respectively. The average pH for treatment A media were significantly (P<0.05) higher than the average for treatment B media (Table 2). The significant differences in pH and the variations in the proportion of vermicast in the different mixed media influenced the composition of nutrients released and the water quality parameters.

**Table 2:** Two-sample independent t-test for comparing electrical conductivity (EC), total dissolved solids (TDS), salinity and pH for vermicast-sawdust mixed media without (A) and with (B) *Trichoderma viride* in the nutrient release experiments.

Growing Medium	pH	EC	TDS	Salinity
**A1**	7.41±0.16	122.4±43.9	84.6±31.5	57.2±20.8
**B1**	7.21±0.23	125.4±56.7	88.6±39.8	59.4±26.9
**P-value**	0.003	ns	ns	ns
**A2**	7.37±0.13	101.8±38.0	71.0±26.2	47.5±17.7
**B2**	7.18±0.23	88.3±41.1	61.9±28.7	41.3±19.5
**P-value**	0.002	ns	ns	ns
**A3**	7.35±0.12	88.7±43.2	62.0±30.2	41.6±20.2
**B3**	7.16±0.21	73.0±33.2	51.4±23.2	34.7±16.1
**P-value**	0.002	ns	ns	ns
**A4**	7.30±0.10	67.4±25.4	47.0±17.9	31.4±12.1
**B4**	7.09±0.19	49.5±21.9	34.6±15.3	23.0±10.2
**P-value**	<0.001	0.026	0.028	0.027
**A5**	7.00±0.20	44.07±9.37	30.99±6.80	20.46±4.48
**B5**	6.72±0.16	30.30±10.50	21.00±7.22	13.91±4.93
**P-value**	<0.001	<0.001	<0.001	<0.001

Values are means ± standard errors and n = 3 per experimental replicate.

ns, no significant difference at P>0.05.

A1, B1 are 80%vermicast+20%sawdust; A2, B2 are 60%vermicast+40%sawdust; A3, B3 are 40%vermicast+60%sawdust; A4, B4 are 20%vermicast+ 80%sawdust; and A5, B5 are sawdust alone.

Therefore, it can be said that the presence of *T*. *viride* might have slowed the release of nutrients probably due to the immobilization of available nutrients. Generally, a considerable portion of nutrients is utilized by microbes for growth, which influenced the chemical properties of the media. The incorporated elemental nutrients become available after the death and degradation of microbial cells [25, 26]. Additionally, *Trichoderma spp* are known to secret organic acids, which decreases soil pH and chelates mineral elements, thus rendering them unavailable for plant uptake [25]. Hence, the mixed media without *T*. *viride*, particularly A1, A2 and A3 will likely be more effective than the media amended with *T*. *viride*.

It was found that unstructured covariance was appropriate for EC, TDS, salinity and pH, and was used in the covariance analysis employed. Multiple means comparison was performed when the repeated measures analysis (RMA) (Proc MIXED) showed a significant (P<0.0001) difference (Table 3). The RMA results confirmed that the interaction effect of Time*Trichoderma*Treatment for TDS was significantly different (P = 0.01). Overall, the multiple means comparison result showed that for each sampling time, the addition of *T*. *viride* increased the values of the water quality parameters for A1 and B1; both of which had the highest proportion of vermicast (S1 Table). For instance, 34.5 hr, with *T*. *viride*, A1 and B1 combination had the highest TDS least-squares mean of 10.18. This could be due to increased nutrient availability with vermicast application and promotion of mineral solubilization by which *Trichoderma* regulate plant nutrient and growth as reported in previous studies [26, 27].

**Table 3:** Repeated measures analysis (RMA) for total dissolved solids, electrical conductivity, salinity and pH for *vermicast-sawdust* mixed media without (A) and with (B) *Trichoderma viride* in the nutrient release experiments.

RMA outcome	Num DF^a^	Den DF	F-Value	P-value
Total dissolved solids				
Time	12	390	361.46	<0.0001
*T*. *viride*	1	390	150.27	<0.0001
Time*T. *viride*	12	390	3.57	<0.0001
Treatment	4	390	414.98	<0.0001
Time*Treatment	48	390	13.94	<0.0001
*T*. viride*Treatment	4	390	8.48	<0.0001
Time*T. viride*Treatment	48	390	1.59	0.0102
Electrical conductivity				
Time	12	390	228.08	<0.0001
*T*. *viride*	1	390	75.97	<0.0001
Time*T. *viride*	12	390	1.57	0.0978
Treatment	4	390	279.63	<0.0001
Time*Treatment	48	390	16.26	<0.0001
*T*. viride*Treatment	4	390	2.23	0.0655
Time*T. viride*Treatment	48	390	1.25	0.1345
Salinity				
Time	12	390	231.42	<0.0001
*T*. *viride*	1	390	70.31	<0.0001
Time*T. *viride*	12	390	2.01	0.0225
Treatment	4	390	303.74	<0.0001
Time*Treatment	48	390	16.76	<0.0001
*T*. viride*Treatment	4	390	3.56	0.0073
Time*T. viride*Treatment	48	390	1.25	0.1325
pH				
Time	12	390	70.08	<0.0001
*T*. *viride*	1	390	3052.65	<0.0001
Time*T. *viride*	12	390	17.46	<0.0001
Treatment	4	390	434.12	<0.0001
Time*Treatment	48	390	27.18	<0.0001
*T*. viride*Treatment	4	390	0.61	0.6530
Time*T. viride*Treatment	48	390	3.83	<0.0001

^a^DF is degrees of freedom; Num DF is numerator DF = k–1; Den DF is denominator DF = N–k; n = 3 per experimental replicate.

However, the lowest TDS least-squares mean was at 0.5 hr, with *T*. *viride*, A5 and B5. The RMA for EC showed a significant (P<0.0001) 2-way interaction of Time *Treatment, and a significant (P<0.0001) main effect of *T*. *viride* (Table 3). For *T*. *viride* main effect on EC, it was confirmed that the media treatments without *T*. *viride* (i.e., group A) had better performance than when *T*. *viride* was added (Table 3). The results indicated that at 34.5 hr, A1 and B1 combination had the highest EC least-squares means (i.e., 141.14). The lowest EC least-squares mean was observed at 0.25 hr, and in A5 and B5 (i.e., 22.67) (S2 Table).

**Fig 3:**
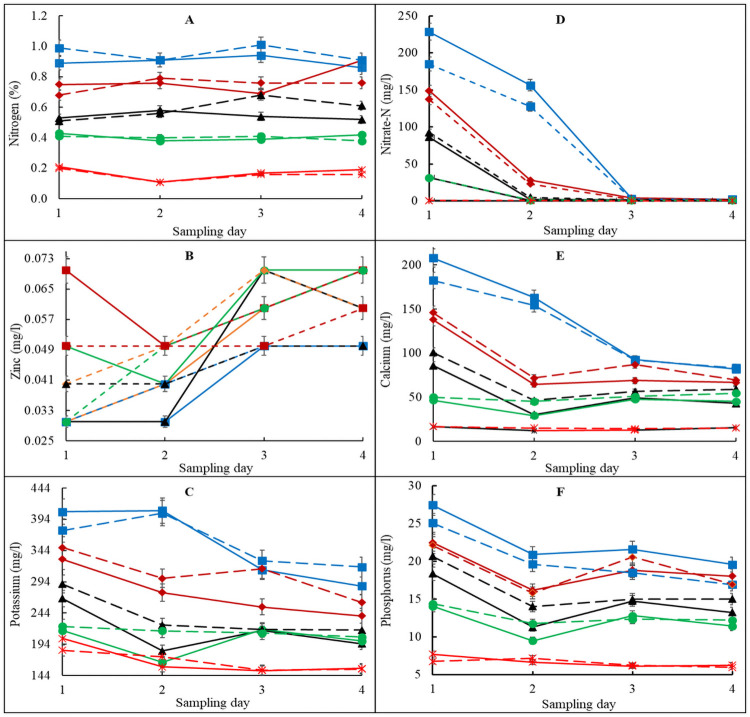
Nitrogen (A), zinc (B), potassium (C), nitrate-nitrogen (D), calcium (E) and phosphorus (F) availability in vermicast-sawdust mixed media without (solid lines) and with (broken lines) addition of *Trichoderma viride* during a 90-day incubation period. A1, B1 (blue line) is 80%vermicast+20%sawdust; A2, B2 (brown line) is 60%vermicast+40%sawdust; A3, B3 (black line) is 40%vermicast+60%sawdust; A4, B4 (green line) is 20%vermicast+ 80%sawdust; and A5, B5 (red line) is sawdust alone. Day 0 (point 1); day 30 (point 2); day 60 (point 3) and day 90 (point 4) with n = 3 per experimental replicate. Error bars represent standard error.

**Fig 4:**
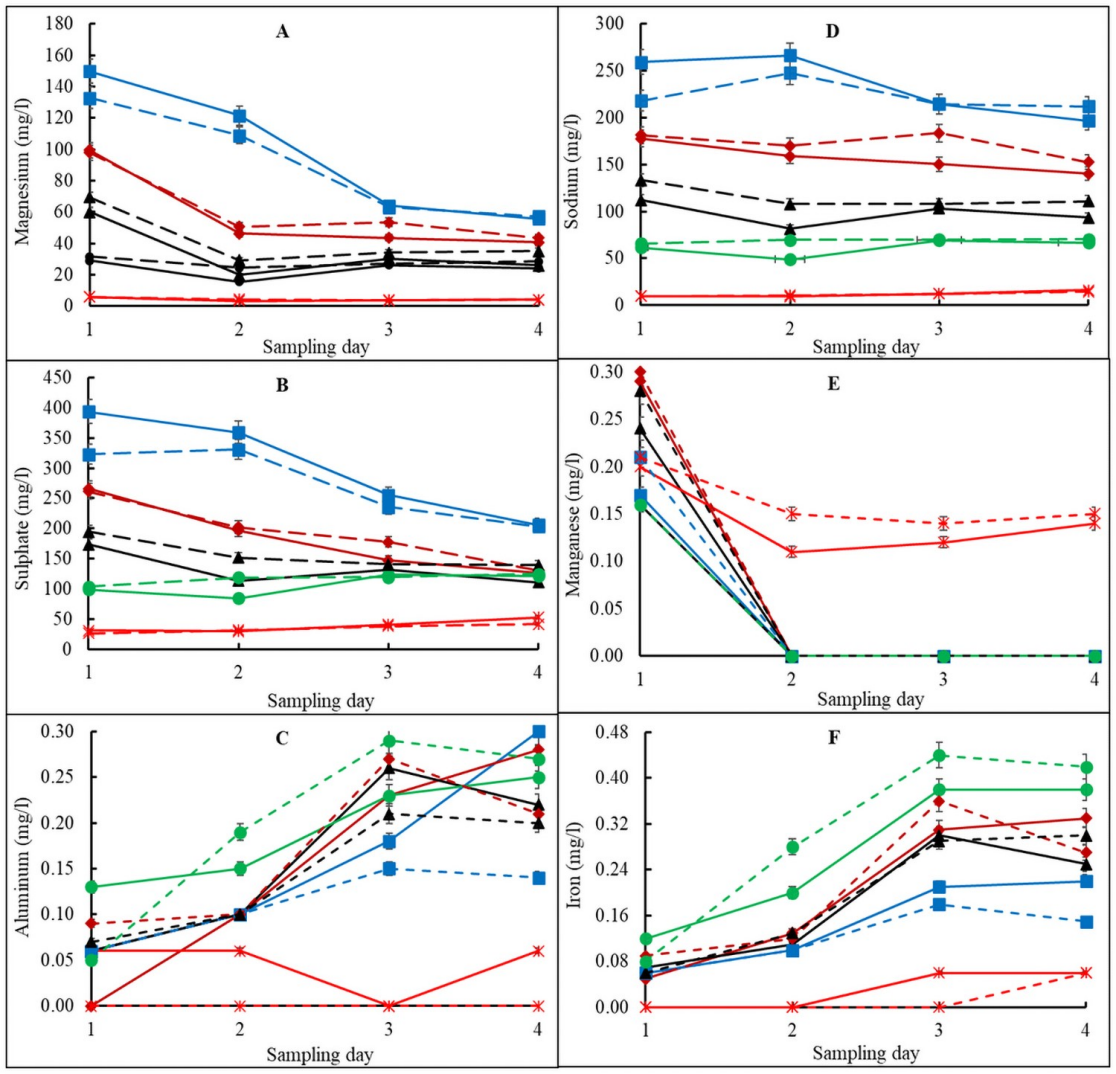
Magnesium (A), sulphate (B), aluminum (C), sodium (D), manganese (E) and iron (F) availability in vermicast-sawdust mixed media without (solid lines) and with (broken lines) addition of *Trichoderma viride* during a 90-day incubation period. A1, B1 (blue line) is 80%vermicast+20%sawdust; A2, B2 (brown line) is 60%vermicast+40%sawdust; A3, B3 (black line) is 40%vermicast+60%sawdust; A4, B4 (green line) is 20%vermicast+ 80%sawdust; and A5, B5 (red line) is sawdust alone. Day 0 (point 1); day 30 (point 2); day 60 (point 3) and day 90 (point 4) with n = 3 per experimental replicate. Error bars represent standard error.

For salinity, all the 2-way analysis showed a significant interactive effect of Time*T. *viride* (P = 0.0225), Time*Treatment (P<0.0001) and *T*. viride*Treatment (P = 0.0073) as presented in S3–S5 Tables. The Time*T. *viride* interaction on salinity revealed that incubation at 34.5 hr, with *T*. *viride* combination had the highest salinity least squares mean (i.e., 43.40), while the lowest salinity least-squares mean was at 0 hr, with *T*. *viride* (i.e., 22.67) (S3 Table). For Time*Treatment interaction on salinity, it was found that at 34.5 hr, A1 and B1 combinations had the highest salinity least squares mean (i.e., 66.51) and the lowest salinity least-squares mean was at 0.25 hr, A5 and B5 (i.e., 10.41) (S4 Table). For *T*. viride*Treatment interaction on salinity, it was found that media without *T*. *viride*, A1 and B1 combinations had the highest salinity least square means (i.e., 47.31), whiles the lowest salinity least-squares mean was observed in *T*. *viride*, A5 and B5 (i.e., 12.11) combinations (S5 Table). Salinity is a major factor affecting media quality and use for crop production. High media salinity results in increased ion toxicity, which is possibly caused by the rapid dissociation of soluble salts [28]. The increase in salts with high vermicast contained media could be attributed to high soluble salt concentration in vermicast as revealed by Joshi and Kelkar [29], They reported that vermicast contains higher soluble salts with higher cation exchangeable properties compared to natural soils. The RMA for pH showed a significant (P<0.0001) 3-way i.e., Time*T. viride*Treatment interaction effect (Table 3). The effect of such interaction on pH showed that 22.5 hrs without *T*. *viride*, A1 and B1 combinations had the highest pH least squares mean (i.e., 7.51). However, the least pH least-squares mean (i.e., 6.62) was recorded in A5 and B5 at 34.5 hr and in A2 and B2 at 2 hr (S6 and S7 Tables). Samples of the incubated mixed media were collected at different times for the determination of nutrients mineralization in the growing media with or without *T*. *viride*. It was evident that the mineralization and composition of mineral nutrients in the different growing media varied with the time of incubation (Figs 3 and 4).

Vermicast is inherently nutrient-rich and commonly used as a growing medium amendment. As such, the growing media with high proportions of vermicast, particularly A1 and B1 had the highest nutrients concentration followed by A2 and B2, A3 and B3, A4 and B4 and the least in A5 and B5. Overall, total N did not change during the incubation period (Fig 3). However, NO_3_^—^N which is a component of total N was reduced to a negligible amount at the 3^rd^ sampling date on day 30 of incubation (Fig 3). These observations suggested N immobilization occurred through bioconversion of NO_3_^—^N to organic forms by microbial populations present in the growing media. It seemed *T*. *viride* did alter N and most of the nutrients in the growing media when group A media was compared to group B media. Zn concentration fluctuated with its reductions in some of the growing media before rising from day 30 and then remained constant in A1, B1 and B2, but increased in A4 and B3 or reduced in A2, B4 and B5 after day 60 (Fig 3). The concentrations of Ca, K and P for A1, A2 and A3; and for B1, B2 and B3 declined at different rates. However, P remained constant after the 2^nd^ sampling date but changes in Ca and K delayed until the 3^rd^ sampling date (Fig 3).

Significant reductions in the concentrations of Mg and SO_4_^2-^ in A1 and B1 occurred followed by moderate reductions in A2 and B2 and slight reductions in A3 and B3 (Fig 4A and 4B).

In general, the patterns of Al and Fe concentrations were similar in groups A and B (Fig 4C and 4F). There were also moderate reductions in the concentrations of Na in treatment A1 and B1 but negligible changes in all the other growing media compositions. This high Na concentration with increased proportion of vermicast was consistent with earlier finding [30], which can potentially affect root nutrient uptake and plant performance (Fig 4D). There was a sharp decline in Mn concentrations in all the growing media except for A5 and B5, which had the highest and stable concentration of Mn (Fig 4E). There was a gentle rise in Al and Fe up to the 3^rd^ sampling point on day 60 before levelling off. In both cases, the concentrations of Al and Fe were moderately higher in B1 than in A1, but they were similar on day 90. It was obvious that concentrations of mineral nutrients in A5 and B5 were the least except for Mn. A4 and B4 were the second worse in mineral nutrients concentration. A5 and B5 were composed of sawdust alone without and with *T*. *viride*, respectively. As a result of the low inherent nutrient concentration, changes in mineral nutrients in A5 and B5 during the 90 days of incubation period were negligible except for Zn.

The findings further confirmed that EC was the best estimator for N and K as treatments with lower EC were also associated with lower N and K concentrations (Table 4). Martínez-Suller et al. [31] reported a high positive correlation between EC and nutrient concentration, which was confirmed by Abbey et al. [19].

**Table 4:** Mineral nutrients concentration of vermicast-sawdust mixed media without (A) and with (B) addition of *Trichoderma viride* during a 90-day incubation period.

Growing Medium	N (%)	EC (μS/cm)	Ca (mg/l)	K (mg/l)	Mg (mg/l)	P (mg/l)	Na (mg/l)	SO_4_^2-^ (mg/l)	Cl^-^ (mg/l)	B (mg/l)
**A1**	0.9a	3400a	136.4a	353.6a	97.8a	22.4a	234.3a	303.7a	405.5a	ND
**A2**	0.8b	2400b	84.7b	274.9b	57.5b	18.9a	156.8b	184.3b	310.0b	ND
**A3**	0.5c	1600bc	52.3bc	215.3bc	34.1bc	14.4b	97.6c	132.7bc	286.0b	0.10b
**A4**	0.4d	1100cd	42.3bc	199.4c	23.8bc	12.0b	61.8d	107.1cd	150.3c	0.13b
**A5**	0.2e	600d	14.4c	166.9c	4.3c	6.7c	12.1e	38.6d	24.0d	0.23a
**B1**	1.0a	3500a	128.2a	356.3a	90.5a	20.0a	223.1a	273.1a	453.3a	ND
**B2**	0.8b	2500b	93.7ab	306.0b	61.4ab	18.9ab	171.9b	193.1b	405.5a	0.10c
**B3**	0.6c	1600c	65.7b	237.2c	42.0bc	16.2bc	115.2c	156.7bc	286.0b	0.11bc
**B4**	0.4d	1100cd	50.3bc	213.9c	27.9cd	12.7c	69.1d	116.5c	107.2c	0.12b
**B5**	0.2e	500d	15.5c	166.3d	4.6d	6.6d	11.8e	34.4d	19.0d	0.25a

Means that do not share the same letter are significantly different at P<0.05. ND, data were not detected; n = 3 per experimental replicate.

A1, B1 are 80%vermicast+20%sawdust; A2, B2 are 60%vermicast+40%sawdust; A3, B3 are 40%vermicast+60%sawdust; A4, B4 are 20%vermicast+ 80%sawdust; and A5, B5 are sawdust alone.

EC is electric conductivity; N is nitrogen; Ca, calcium; K is potassium; Mg is magnesium; P is phosphorus; Na is sodium; SO4_2_^-^ is sulphate; Cl^-^ is chloride; and B is boron.

The deionized water used could conduct electricity due to the presence of charged ions i.e., cations such as Ca^2+^, K^+^ and Mg^2+^ and anions such as NO_3_^-^, SO_4_^2-^ and Cl^-^ released into solution from the growing media treatments. As such, the ionic concentration of mineral elements and compounds as determined by the EC measurements was used to estimate the mineral nutrients content of the growing media. Student t-test confirmed that differences in nutrients concentration between growing medium with *T*. *viride* (group B) and those without *T*. *viride* (group A) were not significant (P>0.05). However, there were significant (P<0.05) differences in mineral nutrients within each group (Table 5) and when the nutrients were released (Figs 3 and 4).

**Table 5:** Least-squares mean of electric conductivity for *T*. *viride* main effect for vermicast-sawdust mixed media without (A) and with (B) *Trichoderma viride* in a nutrient release experiment.

Effect	Estimate	P-value
**Without *T*. *viride***	68.4188	<0.0001
**With *T*. *viride***	56.4762	<0.0001

n = 3 per experimental replicate.

All the analysed mineral nutrients were significantly (P<0.05) highest in A1 and B1 followed by A2 and B2. The least nutrient values were recorded by A5 and B5. Nonetheless, boron (B) was highest in A5 and B5 but was not detected in A1, A2 and B1. Notably, the more the vermicast and the less the incubation time, the better the nutrient status of the growing medium and the more mineral nutrients were available for plant use. Accordingly, the mineral nutrients concentrations of the growing media followed the trend A1, B1 > A2, B2 > A3, B3 > A4, B4 > A5, B5 with an overall average of A > B.

**Fig 5:**
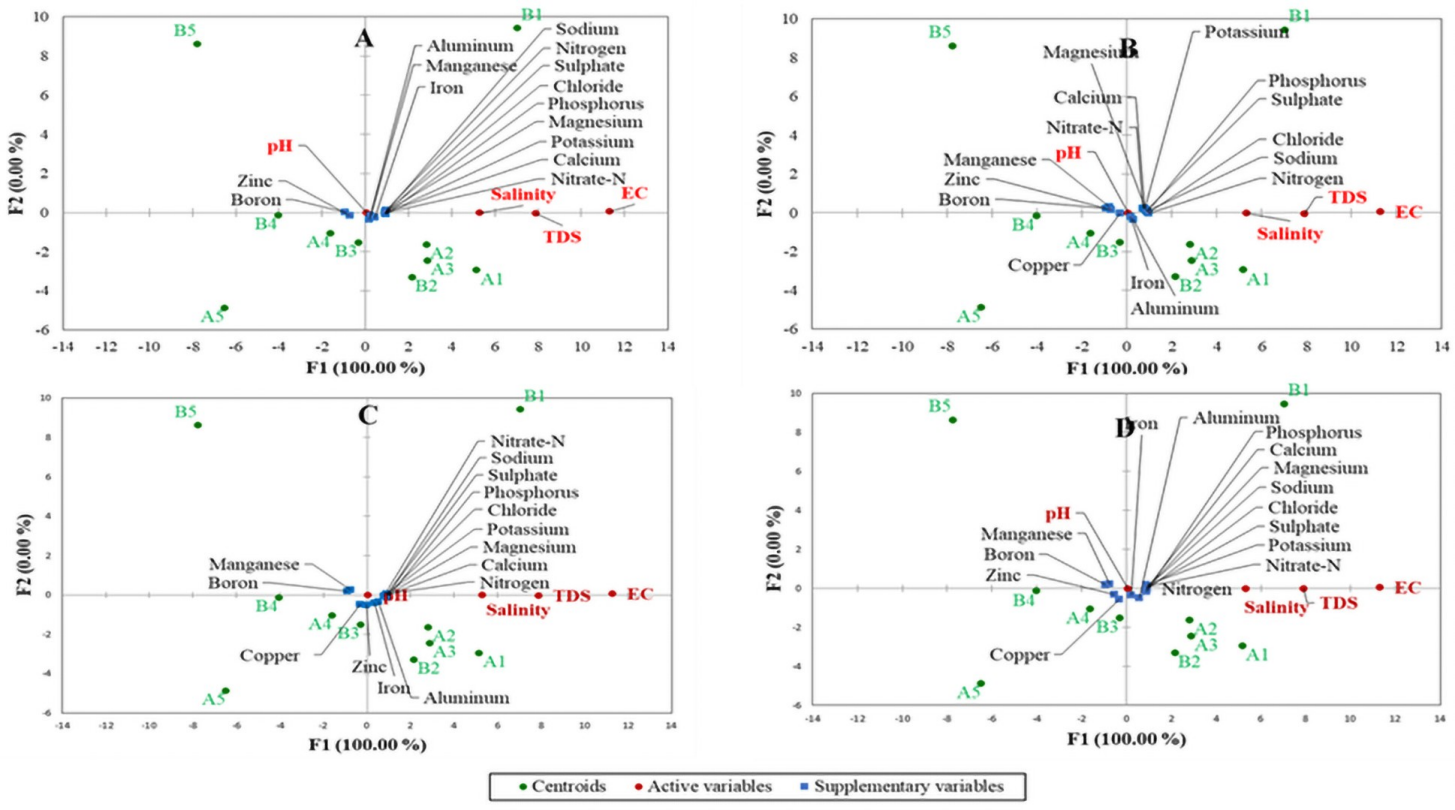
Two-dimension redundancy analyses of growing media nutrient availability and nutrient release as determined using electric conductivity (EC), total dissolved solids (TDS), salinity and pH. A1, B1: 80%vermicast+20%sawdust; A2, B2: 60%vermicast+40%sawdust; A3, B3: 40%vermicast+60%sawdust; A4, B4: 20%vermicast+ 80%sawdust; and A5, B5: sawdust alone. n = 3 per experimental replicate.

A two-dimension redundancy analysis (RDA) was used to further delineate the association amongst the growing media treatments for mineralization and nutrient release (Fig 5A–5D). The RDA showed projections of the variables in the factors space denoted by F1 and F2, which explained 100% of the total variations in the dataset. The RDA explained the existing relationship or otherwise amongst the different growing media, water quality parameters and mineral nutrients. Before incubation for the mineralization experiment, A1, B1, A2, B2, and A3 had a positive and strong relationship with EC, TDS and salinity. These five media were associated with high concentrations of most of the mineral nutrients except for Zn and B, which were high in the other media treatments (Fig 5A). After 30 days of incubation, Mn and Cu were altered and were less associated with A1, A2, A3, and B2 (Fig 5B).

The mineral nutrients distribution on day 60 was like day 1 at the beginning of the incubation, but only Zn and B had a positively high association with B3, A4, B4, A5, and B5 (Fig 5A and 5C). Furthermore, the distribution of mineral nutrients did not change significantly after 90 days of incubation (Fig 5D) as compared to those of days 30 and 60 (Fig 5B and 5C). Overall, A2, A3, B2, B3, A4, and B4 were close to the centre, which suggested that the nutrient release pattern of the media was mainly influenced by the concentrations of the mineral nutrients in the individual media. Although it can be concluded that A1 and B1 had higher mineral nutrients than the other treatments (Figs 4 and 5), and the nutrients released were dependent on concentrations of the nutrients in the media.

## Conclusions

The composition of essential nutrients present in the growing medium is critical to the growth and development of plants and can be estimated using TDS, EC, salinity and pH measurements. We found that nutrients mineralization and availability were higher in sawdust-based media without *T*. *viride* (i.e., treatment A) compared to those with added *T*. *viride* (i.e., treatment B). Hence, *T*. *viride* seems to reduce the rate of media nutrient release and could be used in a slow-release nutrient formulation. The reason is that mineralized nutrients at the end of the studies were similar for individual formulations of A and B. That is, A1 = B1 for the highest nutrient concentration followed by A2 = B2, A3 = B3, A4 = B4 and the least for A5 = B5 in a descending order of magnitude. The interaction of Time*Media Treatment*T. *viride* significantly influenced the nutrient release pattern of the media. Overall, the results showed that vermicast-sawdust mixed media can support plant growth and development. In this case, fast-growing plants may benefit from a growing medium mix of 40% vermicast and 60% sawdust or 60% vermicast and 40% sawdust without *T*. *viride* while slow-growing plants can benefit from the same mixed medium nut with added *T*. *viride*. Further investigation is in progress to assess growing media microbial dynamics and plant growth response.

## Supporting information

S1 Table Treatment combinations. Growing media treatment codes for the mineralization and nutrient-release pattern experiments during December 2018 to February 2019 incubation period.
(DOCX)

S2 Table Least squares mean of total dissolved solids. Determination for Time*Trichoderma viride*Treatment interaction.
(DOCX)

S3 Table Least squares mean of electrical conductivity. Determination for Time*Treatment interaction.
(DOCX)

S4 Table Least squares mean of salinity. Determination for Time*Trichoderma viride interaction.
(DOCX)

S5 Table Least squares mean of salinity. Determination for Time*Treatment interaction.
(DOCX)

S6 Table Least squares mean of salinity. Determination for Trichoderma viride*Treatment interaction.
(DOCX)

S7 Table Least squares mean of pH. Determination for Time*Trichoderma viride*Treatment interaction.
(DOCX)
