# Supplementary material for: Mineralization and nutrient release pattern of vermicast-sawdust mixed media with or without addition of Trichoderma viride
Source: PLoS One. 2021 Jul 8;16(7):e0254188. doi: 10.1371/journal.pone.0254188 (PMC8266104; doi:10.1371/journal.pone.0254188)
Supplement: S1 Table — Growing media treatment codes for the mineralization and nutrient-release pattern experiments during December 2018 to February 2019 incubation period. (DOCX) [file pone.0254188.s001.docx]

S1 Table.

| Code | Vermicast (%) | Sawdust (%) | *T. viride* |
| --- | --- | --- | --- |
| A1 | 80 | 20 | Absent |
| A2 | 60 | 40 | Absent |
| A3 | 40 | 60 | Absent |
| A4 | 20 | 80 | Absent |
| A5 | 0 | 100 | Absent |
| B1 | 80 | 20 | Present |
| B2 | 60 | 40 | Present |
| B3 | 40 | 60 | Present |
| B4 | 20 | 80 | Present |
| B5 | 0 | 100 | Present |

A, no added *T. viride*; B, added *T. viride*.
