# Supplementary material for: Mineralization and nutrient release pattern of vermicast-sawdust mixed media with or without addition of Trichoderma viride
Source: PLoS One. 2021 Jul 8;16(7):e0254188. doi: 10.1371/journal.pone.0254188 (PMC8266104; doi:10.1371/journal.pone.0254188)
Supplement: S2 Table — Determination for Time*Trichoderma viride*Treatment interaction. (DOCX) [file pone.0254188.s002.docx]

S2 Table.

| Effect | Time | *T. viride* | Treatment | Estimate | Estimate |
| --- | --- | --- | --- | --- | --- |
| Time**T. viride**Treatment | 0 | 0 | A1&B1 | 5.44 | <.0001 |
| Time**T. viride**Treatment | 0 | 0 | A2&B2 | 5.03 | <.0001 |
| Time**T. viride**Treatment | 0 | 0 | A3&B3 | 4.56 | <.0001 |
| Time**T. viride**Treatment | 0 | 0 | A4&B4 | 4.51 | <.0001 |
| Time**T. viride**Treatment | 0 | 0 | A5&B5 | 4.50 | <.0001 |
| Time**T. viride**Treatment | 0 | 1 | A1&B1 | 4.19 | <.0001 |
| Time**T. viride**Treatment | 0 | 1 | A2&B2 | 4.00 | <.0001 |
| Time**T. viride**Treatment | 0 | 1 | A3&B3 | 3.89 | <.0001 |
| Time**T. viride**Treatment | 0 | 1 | A4&B4 | 3.64 | <.0001 |
| Time**T. viride**Treatment | 0 | 1 | A5&B5 | 3.36 | <.0001 |
| Time**T. viride**Treatment | 0.25 | 0 | A1&B1 | 5.64 | <.0001 |
| Time**T. viride**Treatment | 0.25 | 0 | A2&B2 | 5.22 | <.0001 |
| Time**T. viride**Treatment | 0.25 | 0 | A3&B3 | 4.68 | <.0001 |
| Time**T. viride**Treatment | 0.25 | 0 | A4&B4 | 4.60 | <.0001 |
| Time**T. viride**Treatment | 0.25 | 0 | A5&B5 | 4.49 | <.0001 |
| Time**T. viride**Treatment | 0.25 | 1 | A1&B1 | 4.23 | <.0001 |
| Time**T. viride**Treatment | 0.25 | 1 | A2&B2 | 4.08 | <.0001 |
| Time**T. viride**Treatment | 0.25 | 1 | A3&B3 | 3.85 | <.0001 |
| Time**T. viride**Treatment | 0.25 | 1 | A4&B4 | 3.55 | <.0001 |
| Time**T. viride**Treatment | 0.25 | 1 | A5&B5 | 3.36 | <.0001 |
| Time**T. viride**Treatment | 0.5 | 0 | A1&B1 | 5.90 | <.0001 |
| Time**T. viride**Treatment | 0.5 | 0 | A2&B2 | 6.08 | <.0001 |
| Time**T. viride**Treatment | 0.5 | 0 | A3&B3 | 5.23 | <.0001 |
| Time**T. viride**Treatment | 0.5 | 0 | A4&B4 | 4.86 | <.0001 |
| Time**T. viride**Treatment | 0.5 | 0 | A5&B5 | 4.62 | <.0001 |
| Time**T. viride**Treatment | 0.5 | 1 | A1&B1 | 5.61 | <.0001 |
| Time**T. viride**Treatment | 0.5 | 1 | A2&B2 | 4.57 | <.0001 |
| Time**T. viride**Treatment | 0.5 | 1 | A3&B3 | 4.18 | <.0001 |
| Time**T. viride**Treatment | 0.5 | 1 | A4&B4 | 3.54 | <.0001 |
| Time**T. viride**Treatment | 0.5 | 1 | A5&B5 | 3.35 | <.0001 |
| Time**T. viride**Treatment | 1 | 0 | A1&B1 | 6.95 | <.0001 |
| Time**T. viride**Treatment | 1 | 0 | A2&B2 | 6.23 | <.0001 |
| Time**T. viride**Treatment | 1 | 0 | A3&B3 | 5.32 | <.0001 |
| Time**T. viride**Treatment | 1 | 0 | A4&B4 | 5.02 | <.0001 |
| Time**T. viride**Treatment | 1 | 0 | A5&B5 | 4.72 | <.0001 |
| Time**T. viride**Treatment | 1 | 1 | A1&B1 | 7.89 | <.0001 |
| Time**T. viride**Treatment | 1 | 1 | A2&B2 | 5.72 | <.0001 |
| Time**T. viride**Treatment | 1 | 1 | A3&B3 | 4.71 | <.0001 |
| Time**T. viride**Treatment | 1 | 1 | A4&B4 | 3.98 | <.0001 |
| Time**T. viride**Treatment | 1 | 1 | A5&B5 | 3.36 | <.0001 |
| Time**T. viride**Treatment | 1.5 | 0 | A1&B1 | 6.69 | <.0001 |
| Time**T. viride**Treatment | 1.5 | 0 | A2&B2 | 6.65 | <.0001 |
| Time**T. viride**Treatment | 1.5 | 0 | A3&B3 | 5.89 | <.0001 |
| Time**T. viride**Treatment | 1.5 | 0 | A4&B4 | 5.52 | <.0001 |
| Time**T. viride**Treatment | 1.5 | 0 | A5&B5 | 4.89 | <.0001 |
| Time**T. viride**Treatment | 1.5 | 1 | A1&B1 | 7.07 | <.0001 |
| Time**T. viride**Treatment | 1.5 | 1 | A2&B2 | 6.08 | <.0001 |
| Time**T. viride**Treatment | 1.5 | 1 | A3&B3 | 5.80 | <.0001 |
| Time**T. viride**Treatment | 1.5 | 1 | A4&B4 | 4.93 | <.0001 |
| Time**T. viride**Treatment | 1.5 | 1 | A5&B5 | 3.78 | <.0001 |
| Time**T. viride**Treatment | 2 | 0 | A1&B1 | 9.23 | <.0001 |
| Time**T. viride**Treatment | 2 | 0 | A2&B2 | 8.02 | <.0001 |
| Time**T. viride**Treatment | 2 | 0 | A3&B3 | 6.07 | <.0001 |
| Time**T. viride**Treatment | 2 | 0 | A4&B4 | 5.96 | <.0001 |
| Time**T. viride**Treatment | 2 | 0 | A5&B5 | 4.91 | <.0001 |
| Time**T. viride**Treatment | 2 | 1 | A1&B1 | 7.73 | <.0001 |
| Time**T. viride**Treatment | 2 | 1 | A2&B2 | 6.62 | <.0001 |
| Time**T. viride**Treatment | 2 | 1 | A3&B3 | 6.15 | <.0001 |
| Time**T. viride**Treatment | 2 | 1 | A4&B4 | 5.35 | <.0001 |
| Time**T. viride**Treatment | 2 | 1 | A5&B5 | 3.84 | <.0001 |
| Time**T. viride**Treatment | 3 | 0 | A1&B1 | 9.15 | <.0001 |
| Time**T. viride**Treatment | 3 | 0 | A2&B2 | 8.50 | <.0001 |
| Time**T. viride**Treatment | 3 | 0 | A3&B3 | 6.68 | <.0001 |
| Time**T. viride**Treatment | 3 | 0 | A4&B4 | 6.39 | <.0001 |
| Time**T. viride**Treatment | 3 | 0 | A5&B5 | 5.39 | <.0001 |
| Time**T. viride**Treatment | 3 | 1 | A1&B1 | 9.53 | <.0001 |
| Time**T. viride**Treatment | 3 | 1 | A2&B2 | 8.04 | <.0001 |
| Time**T. viride**Treatment | 3 | 1 | A3&B3 | 7.53 | <.0001 |
| Time**T. viride**Treatment | 3 | 1 | A4&B4 | 4.09 | <.0001 |
| Time**T. viride**Treatment | 3 | 1 | A5&B5 | 6.09 | <.0001 |
| Time**T. viride**Treatment | 4 | 0 | A1&B1 | 9.10 | <.0001 |
| Time**T. viride**Treatment | 4 | 0 | A2&B2 | 8.32 | <.0001 |
| Time**T. viride**Treatment | 4 | 0 | A3&B3 | 6.98 | <.0001 |
| Time**T. viride**Treatment | 4 | 0 | A4&B4 | 6.54 | <.0001 |
| Time**T. viride**Treatment | 4 | 0 | A5&B5 | 5.53 | <.0001 |
| Time**T. viride**Treatment | 4 | 1 | A1&B1 | 9.90 | <.0001 |
| Time**T. viride**Treatment | 4 | 1 | A2&B2 | 8.10 | <.0001 |
| Time**T. viride**Treatment | 4 | 1 | A3&B3 | 7.22 | <.0001 |
| Time**T. viride**Treatment | 4 | 1 | A4&B4 | 6.25 | <.0001 |
| Time**T. viride**Treatment | 4 | 1 | A5&B5 | 4.34 | <.0001 |
| Time**T. viride**Treatment | 5 | 0 | A1&B1 | 9.49 | <.0001 |
| Time**T. viride**Treatment | 5 | 0 | A2&B2 | 8.57 | <.0001 |
| Time**T. viride**Treatment | 5 | 0 | A3&B3 | 7.38 | <.0001 |
| Time**T. viride**Treatment | 5 | 0 | A4&B4 | 6.78 | <.0001 |
| Time**T. viride**Treatment | 5 | 0 | A5&B5 | 5.66 | <.0001 |
| Time**T. viride**Treatment | 5 | 1 | A1&B1 | 8.91 | <.0001 |
| Time**T. viride**Treatment | 5 | 1 | A2&B2 | 7.37 | <.0001 |
| Time**T. viride**Treatment | 5 | 1 | A3&B3 | 6.98 | <.0001 |
| Time**T. viride**Treatment | 5 | 1 | A4&B4 | 5.90 | <.0001 |
| Time**T. viride**Treatment | 5 | 1 | A5&B5 | 4.44 | <.0001 |
| Time**T. viride**Treatment | 8 | 0 | A1&B1 | 9.71 | <.0001 |
| Time**T. viride**Treatment | 8 | 0 | A2&B2 | 8.73 | <.0001 |
| Time**T. viride**Treatment | 8 | 0 | A3&B3 | 7.90 | <.0001 |
| Time**T. viride**Treatment | 8 | 0 | A4&B4 | 7.00 | <.0001 |
| Time**T. viride**Treatment | 8 | 0 | A5&B5 | 5.79 | <.0001 |
| Time**T. viride**Treatment | 8 | 1 | A1&B1 | 9.07 | <.0001 |
| Time**T. viride**Treatment | 8 | 1 | A2&B2 | 7.41 | <.0001 |
| Time**T. viride**Treatment | 8 | 1 | A3&B3 | 6.88 | <.0001 |
| Time**T. viride**Treatment | 8 | 1 | A4&B4 | 5.85 | <.0001 |
| Time**T. viride**Treatment | 8 | 1 | A5&B5 | 4.52 | <.0001 |
| Time**T. viride**Treatment | 13.5 | 0 | A1&B1 | 9.48 | <.0001 |
| Time**T. viride**Treatment | 13.5 | 0 | A2&B2 | 8.60 | <.0001 |
| Time**T. viride**Treatment | 13.5 | 0 | A3&B3 | 7.92 | <.0001 |
| Time**T. viride**Treatment | 13.5 | 0 | A4&B4 | 6.89 | <.0001 |
| Time**T. viride**Treatment | 13.5 | 0 | A5&B5 | 5.97 | <.0001 |
| Time**T. viride**Treatment | 13.5 | 1 | A1&B1 | 9.37 | <.0001 |
| Time**T. viride**Treatment | 13.5 | 1 | A2&B2 | 7.71 | <.0001 |
| Time**T. viride**Treatment | 13.5 | 1 | A3&B3 | 7.13 | <.0001 |
| Time**T. viride**Treatment | 13.5 | 1 | A4&B4 | 6.00 | <.0001 |
| Time**T. viride**Treatment | 13.5 | 1 | A5&B5 | 4.62 | <.0001 |
| Time**T. viride**Treatment | 22.5 | 0 | A1&B1 | 9.39 | <.0001 |
| Time**T. viride**Treatment | 22.5 | 0 | A2&B2 | 8.40 | <.0001 |
| Time**T. viride**Treatment | 22.5 | 0 | A3&B3 | 8.18 | <.0001 |
| Time**T. viride**Treatment | 22.5 | 0 | A4&B4 | 7.09 | <.0001 |
| Time**T. viride**Treatment | 22.5 | 0 | A5&B5 | 5.90 | <.0001 |
| Time**T. viride**Treatment | 22.5 | 1 | A1&B1 | 9.78 | <.0001 |
| Time**T. viride**Treatment | 22.5 | 1 | A2&B2 | 8.12 | <.0001 |
| Time**T. viride**Treatment | 22.5 | 1 | A3&B3 | 7.40 | <.0001 |
| Time**T. viride**Treatment | 22.5 | 1 | A4&B4 | 6.16 | <.0001 |
| Time**T. viride**Treatment | 22.5 | 1 | A5&B5 | 4.92 | <.0001 |
| Time**T. viride**Treatment | 34.5 | 0 | A1&B1 | 9.57 | <.0001 |
| Time**T. viride**Treatment | 34.5 | 0 | A2&B2 | 8.68 | <.0001 |
| Time**T. viride**Treatment | 34.5 | 0 | A3&B3 | 8.40 | <.0001 |
| Time**T. viride**Treatment | 34.5 | 0 | A4&B4 | 7.18 | <.0001 |
| Time**T. viride**Treatment | 34.5 | 0 | A5&B5 | 5.83 | <.0001 |
| Time**T. viride**Treatment | 34.5 | 1 | A1&B1 | 10.18 | <.0001 |
| Time**T. viride**Treatment | 34.5 | 1 | A2&B2 | 8.42 | <.0001 |
| Time**T. viride**Treatment | 34.5 | 1 | A3&B3 | 7.65 | <.0001 |
| Time**T. viride**Treatment | 34.5 | 1 | A4&B4 | 6.33 | <.0001 |
| Time**T. viride**Treatment | 34.5 | 1 | A5&B5 | 4.88 | <.0001 |

*T. viride* levels, 0 means without it; 1 means with it. A1, 80% vermicast+20% sawdust; A2, 60% vermicast+40% sawdust; A3, 40% vermicast+60% sawdust; A4, 20% vermicast+80% sawdust; A5, sawdust alone (control). The corresponding treatments B1-B5 contained *T. viride*.
