# Supplementary material for: Mineralization and nutrient release pattern of vermicast-sawdust mixed media with or without addition of Trichoderma viride
Source: PLoS One. 2021 Jul 8;16(7):e0254188. doi: 10.1371/journal.pone.0254188 (PMC8266104; doi:10.1371/journal.pone.0254188)
Supplement: S3 Table — Determination for Time*Treatment interaction. (DOCX) [file pone.0254188.s003.docx]

S3 Table.

| Effect | Time | Treatment | Estimate | Estimate |
| --- | --- | --- | --- | --- |
| Time*Treatment | 0 | A1&B1 | 34.73 | <.0001 |
| Time*Treatment | 0 | A2&B2 | 29.61 | <.0001 |
| Time*Treatment | 0 | A3&B3 | 25.79 | <.0001 |
| Time*Treatment | 0 | A4&B4 | 25.48 | <.0001 |
| Time*Treatment | 0 | A5&B5 | 22.91 | <.0001 |
| Time*Treatment | 0.25 | A1&B1 | 35.69 | <.0001 |
| Time*Treatment | 0.25 | A2&B2 | 31.83 | <.0001 |
| Time*Treatment | 0.25 | A3&B3 | 26.54 | <.0001 |
| Time*Treatment | 0.25 | A4&B4 | 23.95 | <.0001 |
| Time*Treatment | 0.25 | A5&B5 | 22.67 | <.0001 |
| Time*Treatment | 0.5 | A1&B1 | 48.33 | <.0001 |
| Time*Treatment | 0.5 | A2&B2 | 41.85 | <.0001 |
| Time*Treatment | 0.5 | A3&B3 | 32.44 | <.0001 |
| Time*Treatment | 0.5 | A4&B4 | 26.05 | <.0001 |
| Time*Treatment | 0.5 | A5&B5 | 23.31 | <.0001 |
| Time*Treatment | 1 | A1&B1 | 80.58 | <.0001 |
| Time*Treatment | 1 | A2&B2 | 51.90 | <.0001 |
| Time*Treatment | 1 | A3&B3 | 36.55 | <.0001 |
| Time*Treatment | 1 | A4&B4 | 29.61 | <.0001 |
| Time*Treatment | 1 | A5&B5 | 24.31 | <.0001 |
| Time*Treatment | 1.5 | A1&B1 | 83.24 | <.0001 |
| Time*Treatment | 1.5 | A2&B2 | 56.49 | <.0001 |
| Time*Treatment | 1.5 | A3&B3 | 48.76 | <.0001 |
| Time*Treatment | 1.5 | A4&B4 | 39.23 | <.0001 |
| Time*Treatment | 1.5 | A5&B5 | 27.53 | <.0001 |
| Time*Treatment | 2 | A1&B1 | 104.35 | <.0001 |
| Time*Treatment | 2 | A2&B2 | 78.18 | <.0001 |
| Time*Treatment | 2 | A3&B3 | 53.74 | <.0001 |
| Time*Treatment | 2 | A4&B4 | 45.96 | <.0001 |
| Time*Treatment | 2 | A5&B5 | 27.91 | <.0001 |
| Time*Treatment | 3 | A1&B1 | 127.95 | <.0001 |
| Time*Treatment | 3 | A2&B2 | 98.86 | <.0001 |
| Time*Treatment | 3 | A3&B3 | 69.29 | <.0001 |
| Time*Treatment | 3 | A4&B4 | 41.51 | <.0001 |
| Time*Treatment | 3 | A5&B5 | 47.21 | <.0001 |
| Time*Treatment | 4 | A1&B1 | 121.70 | <.0001 |
| Time*Treatment | 4 | A2&B2 | 96.81 | <.0001 |
| Time*Treatment | 4 | A3&B3 | 72.36 | <.0001 |
| Time*Treatment | 4 | A4&B4 | 59.24 | <.0001 |
| Time*Treatment | 4 | A5&B5 | 35.66 | <.0001 |
| Time*Treatment | 5 | A1&B1 | 122.18 | <.0001 |
| Time*Treatment | 5 | A2&B2 | 91.99 | <.0001 |
| Time*Treatment | 5 | A3&B3 | 74.25 | <.0001 |
| Time*Treatment | 5 | A4&B4 | 58.25 | <.0001 |
| Time*Treatment | 5 | A5&B5 | 37.19 | <.0001 |
| Time*Treatment | 8 | A1&B1 | 126.89 | <.0001 |
| Time*Treatment | 8 | A2&B2 | 94.09 | <.0001 |
| Time*Treatment | 8 | A3&B3 | 78.75 | <.0001 |
| Time*Treatment | 8 | A4&B4 | 59.70 | <.0001 |
| Time*Treatment | 8 | A5&B5 | 38.48 | <.0001 |
| Time*Treatment | 13.5 | A1&B1 | 124.15 | <.0001 |
| Time*Treatment | 13.5 | A2&B2 | 92.43 | <.0001 |
| Time*Treatment | 13.5 | A3&B3 | 79.50 | <.0001 |
| Time*Treatment | 13.5 | A4&B4 | 58.46 | <.0001 |
| Time*Treatment | 13.5 | A5&B5 | 40.46 | <.0001 |
| Time*Treatment | 22.5 | A1&B1 | 136.31 | <.0001 |
| Time*Treatment | 22.5 | A2&B2 | 98.30 | <.0001 |
| Time*Treatment | 22.5 | A3&B3 | 87.83 | <.0001 |
| Time*Treatment | 22.5 | A4&B4 | 61.88 | <.0001 |
| Time*Treatment | 22.5 | A5&B5 | 41.26 | <.0001 |
| Time*Treatment | 34.5 | A1&B1 | 141.14 | <.0001 |
| Time*Treatment | 34.5 | A2&B2 | 104.72 | <.0001 |
| Time*Treatment | 34.5 | A3&B3 | 93.60 | <.0001 |
| Time*Treatment | 34.5 | A4&B4 | 65.74 | <.0001 |
| Time*Treatment | 34.5 | A5&B5 | 41.46 | <.0001 |

*T. viride* levels, 0 means without it; 1 means with it. A1, 80% vermicast+20% sawdust; A2, 60% vermicast+40% sawdust; A3, 40% vermicast+60% sawdust; A4, 20% vermicast+80% sawdust; A5, sawdust alone (control). The corresponding treatments B1-B5 contained *T. viride*.
