# Supplementary material for: Mineralization and nutrient release pattern of vermicast-sawdust mixed media with or without addition of Trichoderma viride
Source: PLoS One. 2021 Jul 8;16(7):e0254188. doi: 10.1371/journal.pone.0254188 (PMC8266104; doi:10.1371/journal.pone.0254188)
Supplement: S4 Table — Determination for Time*Trichoderma viride interaction. (DOCX) [file pone.0254188.s004.docx]

S4 Table.

| Effect | Time | *T. viride* | Estimate | Estimate |
| --- | --- | --- | --- | --- |
| Time**T. viride* | 0 | 0 | 15.46 | <.0001 |
| Time**T. viride* | 0 | 1 | 9.63 | <.0001 |
| Time**T. viride* | 0.25 | 0 | 16.33 | <.0001 |
| Time**T. viride* | 0.25 | 1 | 9.71 | <.0001 |
| Time**T. viride* | 0.5 | 0 | 19.38 | <.0001 |
| Time**T. viride* | 0.5 | 1 | 12.39 | <.0001 |
| Time**T. viride* | 1 | 0 | 21.98 | <.0001 |
| Time**T. viride* | 1 | 1 | 19.51 | <.0001 |
| Time**T. viride* | 1.5 | 0 | 26.18 | <.0001 |
| Time**T. viride* | 1.5 | 1 | 21.29 | <.0001 |
| Time**T. viride* | 2 | 0 | 33.12 | <.0001 |
| Time**T. viride* | 2 | 1 | 24.72 | <.0001 |
| Time**T. viride* | 3 | 0 | 36.45 | <.0001 |
| Time**T. viride* | 3 | 1 | 35.54 | <.0001 |
| Time**T. viride* | 4 | 0 | 36.76 | <.0001 |
| Time**T. viride* | 4 | 1 | 37.22 | <.0001 |
| Time**T. viride* | 5 | 0 | 39.73 | <.0001 |
| Time**T. viride* | 5 | 1 | 31.87 | <.0001 |
| Time**T. viride* | 8 | 0 | 42.35 | <.0001 |
| Time**T. viride* | 8 | 1 | 32.17 | <.0001 |
| Time**T. viride* | 13.5 | 0 | 41.51 | <.0001 |
| Time**T. viride* | 13.5 | 1 | 32.17 | <.0001 |
| Time**T. viride* | 22.5 | 0 | 41.22 | <.0001 |
| Time**T. viride* | 22.5 | 1 | 39.55 | <.0001 |
| Time**T. viride* | 34.5 | 0 | 43.40 | <.0001 |
| Time**T. viride* | 34.5 | 1 | 40.04 | <.0001 |

*T. viride* levels, 0 means without it; 1 means with it.
