# Supplementary material for: Mineralization and nutrient release pattern of vermicast-sawdust mixed media with or without addition of Trichoderma viride
Source: PLoS One. 2021 Jul 8;16(7):e0254188. doi: 10.1371/journal.pone.0254188 (PMC8266104; doi:10.1371/journal.pone.0254188)
Supplement: S5 Table — Determination for Time*Treatment interaction. (DOCX) [file pone.0254188.s005.docx]

S5 Table.

| Effect | Time | Treatment | Estimate | Estimate |
| --- | --- | --- | --- | --- |
| Time*Treatment | 0 | A1&B1 | 15.65 | <.0001 |
| Time*Treatment | 0 | A2&B2 | 13.61 | <.0001 |
| Time*Treatment | 0 | A3&B3 | 11.90 | <.0001 |
| Time*Treatment | 0 | A4&B4 | 11.03 | <.0001 |
| Time*Treatment | 0 | A5&B5 | 10.53 | <.0001 |
| Time*Treatment | 0.25 | A1&B1 | 16.60 | <.0001 |
| Time*Treatment | 0.25 | A2&B2 | 14.49 | <.0001 |
| Time*Treatment | 0.25 | A3&B3 | 12.21 | <.0001 |
| Time*Treatment | 0.25 | A4&B4 | 11.38 | <.0001 |
| Time*Treatment | 0.25 | A5&B5 | 10.41 | <.0001 |
| Time*Treatment | 0.5 | A1&B1 | 22.36 | <.0001 |
| Time*Treatment | 0.5 | A2&B2 | 19.41 | <.0001 |
| Time*Treatment | 0.5 | A3&B3 | 14.99 | <.0001 |
| Time*Treatment | 0.5 | A4&B4 | 11.93 | <.0001 |
| Time*Treatment | 0.5 | A5&B5 | 10.73 | <.0001 |
| Time*Treatment | 1 | A1&B1 | 37.69 | <.0001 |
| Time*Treatment | 1 | A2&B2 | 24.25 | <.0001 |
| Time*Treatment | 1 | A3&B3 | 16.90 | <.0001 |
| Time*Treatment | 1 | A4&B4 | 13.71 | <.0001 |
| Time*Treatment | 1 | A5&B5 | 11.18 | <.0001 |
| Time*Treatment | 1.5 | A1&B1 | 38.15 | <.0001 |
| Time*Treatment | 1.5 | A2&B2 | 26.91 | <.0001 |
| Time*Treatment | 1.5 | A3&B3 | 22.94 | <.0001 |
| Time*Treatment | 1.5 | A4&B4 | 18.14 | <.0001 |
| Time*Treatment | 1.5 | A5&B5 | 12.53 | <.0001 |
| Time*Treatment | 2 | A1&B1 | 49.23 | <.0001 |
| Time*Treatment | 2 | A2&B2 | 35.95 | <.0001 |
| Time*Treatment | 2 | A3&B3 | 25.35 | <.0001 |
| Time*Treatment | 2 | A4&B4 | 21.28 | <.0001 |
| Time*Treatment | 2 | A5&B5 | 12.79 | <.0001 |
| Time*Treatment | 3 | A1&B1 | 60.03 | <.0001 |
| Time*Treatment | 3 | A2&B2 | 46.21 | <.0001 |
| Time*Treatment | 3 | A3&B3 | 32.35 | <.0001 |
| Time*Treatment | 3 | A4&B4 | 19.31 | <.0001 |
| Time*Treatment | 3 | A5&B5 | 22.05 | <.0001 |
| Time*Treatment | 4 | A1&B1 | 61.95 | <.0001 |
| Time*Treatment | 4 | A2&B2 | 45.23 | <.0001 |
| Time*Treatment | 4 | A3&B3 | 33.94 | <.0001 |
| Time*Treatment | 4 | A4&B4 | 27.34 | <.0001 |
| Time*Treatment | 4 | A5&B5 | 16.50 | <.0001 |
| Time*Treatment | 5 | A1&B1 | 57.45 | <.0001 |
| Time*Treatment | 5 | A2&B2 | 42.83 | <.0001 |
| Time*Treatment | 5 | A3&B3 | 34.45 | <.0001 |
| Time*Treatment | 5 | A4&B4 | 27.05 | <.0001 |
| Time*Treatment | 5 | A5&B5 | 17.21 | <.0001 |
| Time*Treatment | 8 | A1&B1 | 59.55 | <.0001 |
| Time*Treatment | 8 | A2&B2 | 44.11 | <.0001 |
| Time*Treatment | 8 | A3&B3 | 36.89 | <.0001 |
| Time*Treatment | 8 | A4&B4 | 27.83 | <.0001 |
| Time*Treatment | 8 | A5&B5 | 17.91 | <.0001 |
| Time*Treatment | 13.5 | A1&B1 | 58.16 | <.0001 |
| Time*Treatment | 13.5 | A2&B2 | 43.29 | <.0001 |
| Time*Treatment | 13.5 | A3&B3 | 37.08 | <.0001 |
| Time*Treatment | 13.5 | A4&B4 | 27.10 | <.0001 |
| Time*Treatment | 13.5 | A5&B5 | 18.58 | <.0001 |
| Time*Treatment | 22.5 | A1&B1 | 62.28 | <.0001 |
| Time*Treatment | 22.5 | A2&B2 | 45.90 | <.0001 |
| Time*Treatment | 22.5 | A3&B3 | 45.94 | <.0001 |
| Time*Treatment | 22.5 | A4&B4 | 28.86 | <.0001 |
| Time*Treatment | 22.5 | A5&B5 | 18.94 | <.0001 |
| Time*Treatment | 34.5 | A1&B1 | 66.51 | <.0001 |
| Time*Treatment | 34.5 | A2&B2 | 48.94 | <.0001 |
| Time*Treatment | 34.5 | A3&B3 | 43.55 | <.0001 |
| Time*Treatment | 34.5 | A4&B4 | 30.78 | <.0001 |
| Time*Treatment | 34.5 | A5&B5 | 18.81 | <.0001 |

A1, 80% vermicast+20% sawdust; A2, 60% vermicast+40% sawdust; A3, 40% vermicast+60% sawdust; A4, 20% vermicast+80% sawdust; A5, sawdust alone (control). The corresponding treatments B1-B5 contained *T. viride*.
