# Supplementary material for: Mineralization and nutrient release pattern of vermicast-sawdust mixed media with or without addition of Trichoderma viride
Source: PLoS One. 2021 Jul 8;16(7):e0254188. doi: 10.1371/journal.pone.0254188 (PMC8266104; doi:10.1371/journal.pone.0254188)
Supplement: S6 Table — Determination for Trichoderma viride*Treatment interaction. (DOCX) [file pone.0254188.s006.docx]

S6 Table.

| Effect | *T. viride* | Treatment | Estimate | Estimate |
| --- | --- | --- | --- | --- |
| *T. viride**Treatment | 0 | A1&B1 | 47.31 | <.0001 |
| *T. viride**Treatment | 0 | A2&B2 | 38.55 | <.0001 |
| *T. viride**Treatment | 0 | A3&B3 | 30.15 | <.0001 |
| *T. viride**Treatment | 0 | A4&B4 | 24.79 | <.0001 |
| *T. viride**Treatment | 0 | A5&B5 | 18.37 | <.0001 |
| *T. viride**Treatment | 1 | A1&B1 | 45.86 | <.0001 |
| *T. viride**Treatment | 1 | A2&B2 | 30.85 | <.0001 |
| *T. viride**Treatment | 1 | A3&B3 | 26.54 | <.0001 |
| *T. viride**Treatment | 1 | A4&B4 | 17.63 | <.0001 |
| *T. viride**Treatment | 1 | A5&B5 | 12.11 | <.0001 |

*T. viride* levels, 0 means without *T. viride*; 1 means with *T. viride*. A1, 80% vermicast+20% sawdust; A2, 60% vermicast+40% sawdust; A3, 40% vermicast+60% sawdust; A4, 20% vermicast+80% sawdust; A5, sawdust alone (control). The corresponding treatments B1-B5 contained *T. viride*.
