# Supplementary material for: Mineralization and nutrient release pattern of vermicast-sawdust mixed media with or without addition of Trichoderma viride
Source: PLoS One. 2021 Jul 8;16(7):e0254188. doi: 10.1371/journal.pone.0254188 (PMC8266104; doi:10.1371/journal.pone.0254188)
Supplement: S7 Table — Determination for Time*Trichoderma viride*Treatment interaction. (DOCX) [file pone.0254188.s007.docx]

S7 Table.

| Effect | Time | *T. viride* | Treatment | Estimate | Estimate |
| --- | --- | --- | --- | --- | --- |
| Time**T. viride**Treatment | 0 | 0 | A1&B1 | 7.25 | <.0001 |
| Time**T. viride**Treatment | 0 | 0 | A2&B2 | 7.21 | <.0001 |
| Time**T. viride**Treatment | 0 | 0 | A3&B3 | 7.26 | <.0001 |
| Time**T. viride**Treatment | 0 | 0 | A4&B4 | 7.21 | <.0001 |
| Time**T. viride**Treatment | 0 | 0 | A5&B5 | 7.20 | <.0001 |
| Time**T. viride**Treatment | 0 | 1 | A1&B1 | 6.81 | <.0001 |
| Time**T. viride**Treatment | 0 | 1 | A2&B2 | 6.82 | <.0001 |
| Time**T. viride**Treatment | 0 | 1 | A3&B3 | 6.79 | <.0001 |
| Time**T. viride**Treatment | 0 | 1 | A4&B4 | 6.77 | <.0001 |
| Time**T. viride**Treatment | 0 | 1 | A5&B5 | 6.74 | <.0001 |
| Time**T. viride**Treatment | 0.25 | 0 | A1&B1 | 7.19 | <.0001 |
| Time**T. viride**Treatment | 0.25 | 0 | A2&B2 | 7.21 | <.0001 |
| Time**T. viride**Treatment | 0.25 | 0 | A3&B3 | 7.17 | <.0001 |
| Time**T. viride**Treatment | 0.25 | 0 | A4&B4 | 7.26 | <.0001 |
| Time**T. viride**Treatment | 0.25 | 0 | A5&B5 | 7.19 | <.0001 |
| Time**T. viride**Treatment | 0.25 | 1 | A1&B1 | 6.92 | <.0001 |
| Time**T. viride**Treatment | 0.25 | 1 | A2&B2 | 6.88 | <.0001 |
| Time**T. viride**Treatment | 0.25 | 1 | A3&B3 | 6.91 | <.0001 |
| Time**T. viride**Treatment | 0.25 | 1 | A4&B4 | 6.90 | <.0001 |
| Time**T. viride**Treatment | 0.25 | 1 | A5&B5 | 6.85 | <.0001 |
| Time**T. viride**Treatment | 0.5 | 0 | A1&B1 | 7.26 | <.0001 |
| Time**T. viride**Treatment | 0.5 | 0 | A2&B2 | 7.23 | <.0001 |
| Time**T. viride**Treatment | 0.5 | 0 | A3&B3 | 7.20 | <.0001 |
| Time**T. viride**Treatment | 0.5 | 0 | A4&B4 | 7.19 | <.0001 |
| Time**T. viride**Treatment | 0.5 | 0 | A5&B5 | 7.19 | <.0001 |
| Time**T. viride**Treatment | 0.5 | 1 | A1&B1 | 7.00 | <.0001 |
| Time**T. viride**Treatment | 0.5 | 1 | A2&B2 | 6.98 | <.0001 |
| Time**T. viride**Treatment | 0.5 | 1 | A3&B3 | 7.00 | <.0001 |
| Time**T. viride**Treatment | 0.5 | 1 | A4&B4 | 6.95 | <.0001 |
| Time**T. viride**Treatment | 0.5 | 1 | A5&B5 | 6.94 | <.0001 |
| Time**T. viride**Treatment | 1 | 0 | A1&B1 | 7.24 | <.0001 |
| Time**T. viride**Treatment | 1 | 0 | A2&B2 | 7.23 | <.0001 |
| Time**T. viride**Treatment | 1 | 0 | A3&B3 | 7.24 | <.0001 |
| Time**T. viride**Treatment | 1 | 0 | A4&B4 | 7.17 | <.0001 |
| Time**T. viride**Treatment | 1 | 0 | A5&B5 | 7.20 | <.0001 |
| Time**T. viride**Treatment | 1 | 1 | A1&B1 | 7.06 | <.0001 |
| Time**T. viride**Treatment | 1 | 1 | A2&B2 | 7.02 | <.0001 |
| Time**T. viride**Treatment | 1 | 1 | A3&B3 | 6.98 | <.0001 |
| Time**T. viride**Treatment | 1 | 1 | A4&B4 | 7.00 | <.0001 |
| Time**T. viride**Treatment | 1 | 1 | A5&B5 | 6.90 | <.0001 |
| Time**T. viride**Treatment | 1.5 | 0 | A1&B1 | 7.20 | <.0001 |
| Time**T. viride**Treatment | 1.5 | 0 | A2&B2 | 7.22 | <.0001 |
| Time**T. viride**Treatment | 1.5 | 0 | A3&B3 | 7.26 | <.0001 |
| Time**T. viride**Treatment | 1.5 | 0 | A4&B4 | 7.20 | <.0001 |
| Time**T. viride**Treatment | 1.5 | 0 | A5&B5 | 7.19 | <.0001 |
| Time**T. viride**Treatment | 1.5 | 1 | A1&B1 | 7.01 | <.0001 |
| Time**T. viride**Treatment | 1.5 | 1 | A2&B2 | 6.95 | <.0001 |
| Time**T. viride**Treatment | 1.5 | 1 | A3&B3 | 6.97 | <.0001 |
| Time**T. viride**Treatment | 1.5 | 1 | A4&B4 | 6.96 | <.0001 |
| Time**T. viride**Treatment | 1.5 | 1 | A5&B5 | 6.83 | <.0001 |
| Time**T. viride**Treatment | 2 | 0 | A1&B1 | 7.23 | <.0001 |
| Time**T. viride**Treatment | 2 | 0 | A2&B2 | 7.27 | <.0001 |
| Time**T. viride**Treatment | 2 | 0 | A3&B3 | 7.20 | <.0001 |
| Time**T. viride**Treatment | 2 | 0 | A4&B4 | 7.16 | <.0001 |
| Time**T. viride**Treatment | 2 | 0 | A5&B5 | 7.01 | <.0001 |
| Time**T. viride**Treatment | 2 | 1 | A1&B1 | 7.02 | <.0001 |
| Time**T. viride**Treatment | 2 | 1 | A2&B2 | 6.98 | <.0001 |
| Time**T. viride**Treatment | 2 | 1 | A3&B3 | 6.98 | <.0001 |
| Time**T. viride**Treatment | 2 | 1 | A4&B4 | 6.90 | <.0001 |
| Time**T. viride**Treatment | 2 | 1 | A5&B5 | 6.80 | <.0001 |
| Time**T. viride**Treatment | 3 | 0 | A1&B1 | 7.34 | <.0001 |
| Time**T. viride**Treatment | 3 | 0 | A2&B2 | 7.32 | <.0001 |
| Time**T. viride**Treatment | 3 | 0 | A3&B3 | 7.30 | <.0001 |
| Time**T. viride**Treatment | 3 | 0 | A4&B4 | 7.28 | <.0001 |
| Time**T. viride**Treatment | 3 | 0 | A5&B5 | 7.04 | <.0001 |
| Time**T. viride**Treatment | 3 | 1 | A1&B1 | 7.07 | <.0001 |
| Time**T. viride**Treatment | 3 | 1 | A2&B2 | 7.08 | <.0001 |
| Time**T. viride**Treatment | 3 | 1 | A3&B3 | 7.05 | <.0001 |
| Time**T. viride**Treatment | 3 | 1 | A4&B4 | 6.82 | <.0001 |
| Time**T. viride**Treatment | 3 | 1 | A5&B5 | 7.00 | <.0001 |
| Time**T. viride**Treatment | 4 | 0 | A1&B1 | 7.38 | <.0001 |
| Time**T. viride**Treatment | 4 | 0 | A2&B2 | 7.39 | <.0001 |
| Time**T. viride**Treatment | 4 | 0 | A3&B3 | 7.36 | <.0001 |
| Time**T. viride**Treatment | 4 | 0 | A4&B4 | 7.29 | <.0001 |
| Time**T. viride**Treatment | 4 | 0 | A5&B5 | 7.07 | <.0001 |
| Time**T. viride**Treatment | 4 | 1 | A1&B1 | 7.15 | <.0001 |
| Time**T. viride**Treatment | 4 | 1 | A2&B2 | 7.12 | <.0001 |
| Time**T. viride**Treatment | 4 | 1 | A3&B3 | 7.05 | <.0001 |
| Time**T. viride**Treatment | 4 | 1 | A4&B4 | 7.02 | <.0001 |
| Time**T. viride**Treatment | 4 | 1 | A5&B5 | 6.78 | <.0001 |
| Time**T. viride**Treatment | 5 | 0 | A1&B1 | 7.34 | <.0001 |
| Time**T. viride**Treatment | 5 | 0 | A2&B2 | 7.29 | <.0001 |
| Time**T. viride**Treatment | 5 | 0 | A3&B3 | 7.31 | <.0001 |
| Time**T. viride**Treatment | 5 | 0 | A4&B4 | 7.28 | <.0001 |
| Time**T. viride**Treatment | 5 | 0 | A5&B5 | 7.16 | <.0001 |
| Time**T. viride**Treatment | 5 | 1 | A1&B1 | 7.16 | <.0001 |
| Time**T. viride**Treatment | 5 | 1 | A2&B2 | 7.14 | <.0001 |
| Time**T. viride**Treatment | 5 | 1 | A3&B3 | 7.10 | <.0001 |
| Time**T. viride**Treatment | 5 | 1 | A4&B4 | 7.02 | <.0001 |
| Time**T. viride**Treatment | 5 | 1 | A5&B5 | 6.82 | <.0001 |
| Time**T. viride**Treatment | 8 | 0 | A1&B1 | 7.38 | <.0001 |
| Time**T. viride**Treatment | 8 | 0 | A2&B2 | 7.37 | <.0001 |
| Time**T. viride**Treatment | 8 | 0 | A3&B3 | 7.33 | <.0001 |
| Time**T. viride**Treatment | 8 | 0 | A4&B4 | 7.26 | <.0001 |
| Time**T. viride**Treatment | 8 | 0 | A5&B5 | 6.94 | <.0001 |
| Time**T. viride**Treatment | 8 | 1 | A1&B1 | 7.24 | <.0001 |
| Time**T. viride**Treatment | 8 | 1 | A2&B2 | 7.20 | <.0001 |
| Time**T. viride**Treatment | 8 | 1 | A3&B3 | 7.15 | <.0001 |
| Time**T. viride**Treatment | 8 | 1 | A4&B4 | 7.09 | <.0001 |
| Time**T. viride**Treatment | 8 | 1 | A5&B5 | 6.79 | <.0001 |
| Time**T. viride**Treatment | 13.5 | 0 | A1&B1 | 7.44 | <.0001 |
| Time**T. viride**Treatment | 13.5 | 0 | A2&B2 | 7.43 | <.0001 |
| Time**T. viride**Treatment | 13.5 | 0 | A3&B3 | 7.33 | <.0001 |
| Time**T. viride**Treatment | 13.5 | 0 | A4&B4 | 7.25 | <.0001 |
| Time**T. viride**Treatment | 13.5 | 0 | A5&B5 | 6.88 | <.0001 |
| Time**T. viride**Treatment | 13.5 | 1 | A1&B1 | 7.24 | <.0001 |
| Time**T. viride**Treatment | 13.5 | 1 | A2&B2 | 7.20 | <.0001 |
| Time**T. viride**Treatment | 13.5 | 1 | A3&B3 | 7.15 | <.0001 |
| Time**T. viride**Treatment | 13.5 | 1 | A4&B4 | 7.09 | <.0001 |
| Time**T. viride**Treatment | 13.5 | 1 | A5&B5 | 6.79 | <.0001 |
| Time**T. viride**Treatment | 22.5 | 0 | A1&B1 | 7.51 | <.0001 |
| Time**T. viride**Treatment | 22.5 | 0 | A2&B2 | 7.46 | <.0001 |
| Time**T. viride**Treatment | 22.5 | 0 | A3&B3 | 7.42 | <.0001 |
| Time**T. viride**Treatment | 22.5 | 0 | A4&B4 | 7.34 | <.0001 |
| Time**T. viride**Treatment | 22.5 | 0 | A5&B5 | 6.94 | <.0001 |
| Time**T. viride**Treatment | 22.5 | 1 | A1&B1 | 7.25 | <.0001 |
| Time**T. viride**Treatment | 22.5 | 1 | A2&B2 | 7.31 | <.0001 |
| Time**T. viride**Treatment | 22.5 | 1 | A3&B3 | 7.30 | <.0001 |
| Time**T. viride**Treatment | 22.5 | 1 | A4&B4 | 7.21 | <.0001 |
| Time**T. viride**Treatment | 22.5 | 1 | A5&B5 | 6.72 | <.0001 |
| Time**T. viride**Treatment | 34.5 | 0 | A1&B1 | 7.43 | <.0001 |
| Time**T. viride**Treatment | 34.5 | 0 | A2&B2 | 7.38 | <.0001 |
| Time**T. viride**Treatment | 34.5 | 0 | A3&B3 | 7.35 | <.0001 |
| Time**T. viride**Treatment | 34.5 | 0 | A4&B4 | 7.32 | <.0001 |
| Time**T. viride**Treatment | 34.5 | 0 | A5&B5 | 6.90 | <.0001 |
| Time**T. viride**Treatment | 34.5 | 1 | A1&B1 | 7.10 | <.0001 |
| Time**T. viride**Treatment | 34.5 | 1 | A2&B2 | 7.02 | <.0001 |
| Time**T. viride**Treatment | 34.5 | 1 | A3&B3 | 7.16 | <.0001 |
| Time**T. viride**Treatment | 34.5 | 1 | A4&B4 | 7.14 | <.0001 |
| Time**T. viride**Treatment | 34.5 | 1 | A5&B5 | 6.62 | <.0001 |

*T. viride* levels, 0 means without it; 1 means with it. A1, 80% vermicast+20% sawdust; A2, 60% vermicast+40% sawdust; A3, 40% vermicast+60% sawdust; A4, 20% vermicast+80% sawdust; A5, sawdust alone (control). The corresponding treatments B1-B5 contained *T. viride*.
